# A novel prognostic prediction model for recurrence in patients with stage II colon cancer after curative resection

**DOI:** 10.3892/mco.2019.1879

**Published:** 2019-06-13

**Authors:** Kazuhiro Saso, Norikatsu Myoshi, Shiki Fujino, Yuya Takenaka, Yusuke Takahashi, Junichi Nishimura, Masayoshi Yasui, Masayuki Ohue, Masayoshi Tokuoka, Yoshihito Ide, Hidekazu Takahashi, Naotsugu Haraguchi, Taishi Hata, Chu Matsuda, Tsunekazu Mizushima, Yuichiro Doki, Masaki Mori

Mol Clin Oncol 9: 697-701, 2018; DOI: 10.3892/mco.2018.1733

Subsequently to the publication of this article, the authors have realized that the name of the tenth listed author was spelt incorrectly. The name was spelt as ‘Yoshito Ide’, whereas the correct spelling should have been ‘Yoshi**hi**to Ide’ (as shown above).

The authors regret that this error was not recognized and corrected prior to the publication of the above article, and apologize for any inconvenience caused.

